# Acute Ulnar Neuropathy at the Elbow: Clinical, Electrodiagnostic, and Ultrasonographic Findings

**DOI:** 10.3390/neurolint18080152

**Published:** 2026-08-18

**Authors:** Vasudeva G. Iyer, Lisa B. E. Shields, Smita Ghare, Christopher B. Shields

**Affiliations:** 1 Neurodiagnostic Center of Louisville, Louisville, KY 40245, USA; pavaiyer@gmail.com; 2 Norton Neuroscience Institute, Norton Healthcare, 210 East Gray Street, Suite 1102, Louisville, KY 40202, USA; LBES@earthlink.net (L.B.E.S.); smita.ghare@nortonhealthcare.org (S.G.)

**Keywords:** acute ulnar neuropathy, elbow, cubital tunnel syndrome, nerve conduction, EMG, ultrasound

## Abstract

Background/Objectives: Ulnar nerve neuropathy at the elbow (UNE) is the second most common focal neuropathy of the upper extremity and often results from entrapment of the ulnar nerve as it traverses the elbow with insidious onset and gradual progression. This study describes the clinical, electrodiagnostic (EDX), and ultrasound (US) findings in a cohort of patients presenting with acute UNE. Methods: This is a review of 103 patients with clinical features of UNE with acute onset who underwent EDX and US studies over a 25-year period (2010–2026) at our Neurodiagnostic Center. Results: Of the 103 patients with acute UNE, all experienced paresthesia in the ulnar nerve distribution. A total of 27 (26.2%) patients had elbow pain, and 99 (96.1%) had decreased pinprick sensation in the ulnar nerve distribution. Weakness of the following muscles was detected: abductor digiti minimi (ADM) (94 [91.3%]), FDI (first dorsal interosseous) (91 [88.3%]), and FDPu (flexor digitorum profundus—ulnar) (76 [73.8%]). Most patients had a clinical grade of 3 (severe: sensory and motor abnormalities as well as atrophy of the FDI and ADM muscles) (93 [90.3%]). Perioperative injuries were the most common etiology of acute UNE, consisting of 65 (63.1%) patients. The perioperative injury was noted after surgical procedures at varied locations, most frequently at the shoulder in 23 (35.4%) followed by coronary artery bypass in 9 (13.8%) and knee joint surgery in 8 (12.3%). Non-iatrogenic injuries affected 11 (10.7%) patients. The US study revealed cysts and tumors such as a Schwannoma in 14/76 (18.4%) patients. Focal demyelination was observed by EDX studies in 95 (92.2%) patients, and a conduction block was detected in 24 (23.3%). Motor and sensory axonal involvement was identified in 81 (78.6%) and 86 (83.5%) patients, respectively. Of the 76 patients who underwent an US study, 68 (90.6%) had an increase in the cross-sectional area of the ulnar nerve and 51 (68.0%) had a hypoechoic ulnar nerve at the elbow. Conclusions: In this study, the most common cause of acute UNE was periprocedural injury, most frequently following shoulder surgery. Acute UNE may also occur without a history of antecedent trauma or a surgical procedure. EDX and US studies provide complementary data to determine the severity and the etiology of acute UNE and guide further management.

## 1. Introduction

Ulnar nerve neuropathy at the elbow (UNE) is the second most common focal neuropathy of the upper extremity following median nerve entrapment at the carpal tunnel [1,2,3,4,5,6,7,8]. UNE has a population incidence of approximately 3 in 10,000 person-years and a prevalence of 6% [2,4]. The mechanism of UNE may be attributed to two factors: (1) the superficial location of the ulnar nerve at the elbow predisposes to extrinsic compression and injuries and (2) the several potential sites of entrapment as the ulnar nerve courses across the elbow from the distal upper arm to the forearm. The most frequent site of entrapment is the cubital tunnel, located between the medial epicondyle and the olecranon process roofed by Osborne’s ligament [1,3,6,8]. The ulnar nerve may also be entrapped more proximally at the medial intermuscular septum and Arcade of Struthers and distally between the heads of the flexor carpi ulnaris (FCU) muscle and the flexor–pronator aponeurosis. Precise localization to one of these specific sites by clinical examination based on the topography of muscle weakness and sensory loss may be challenging. The term cubital tunnel syndrome (CBTS) is often utilized irrespective of these individual locations of involvement across the elbow [8]. The term ulnar neuropathy at the elbow (UNE) is preferred as it describes the situation more correctly. The most common locations of UNE are just proximal to the medial epicondyle (ME) in the retroepicondylar (RTC) groove (80–85% of affected arms) and just distal to the ME under the humeroulnar aponeurotic arcade (HUA) (cubital tunnel) (15–20% of affected arms) [9].

Electrodiagnostic (EDX) evaluation using the short segment study (“inching” study) may be helpful in precisely localizing the site of the compression/injury, especially if there is a conduction block at one of the several sites of UNE [1,2,3,6,8,10,11]. An ultrasound (US) study may be helpful for localization by detecting an increased cross-sectional area (CSA) of the ulnar nerve at different locations around the elbow [2,3,8]. US may also potentially reveal an underlying cause of the ulnar neuropathy [8].

**Figure 1:**
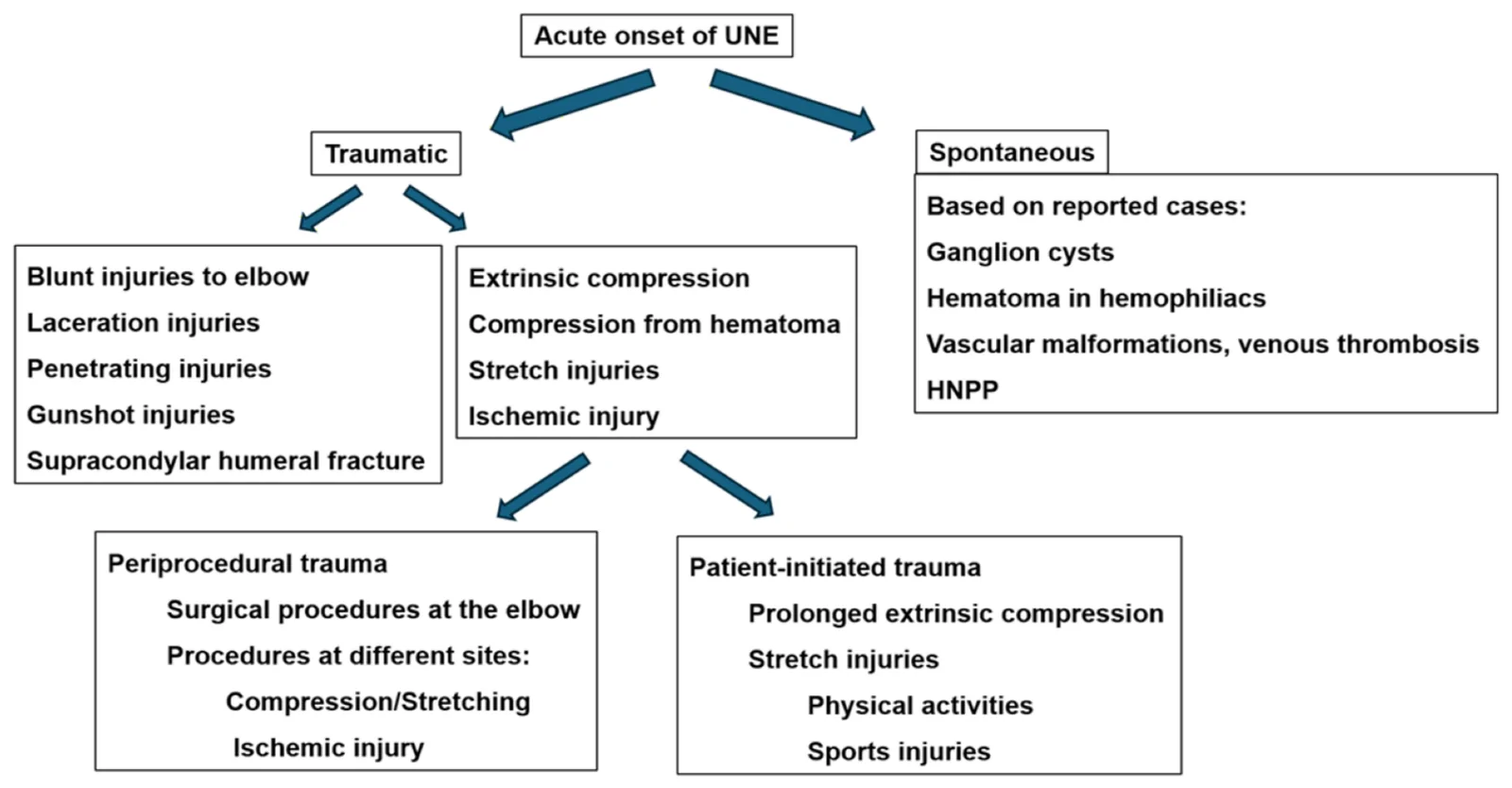
Diagram depicting the mechanism of acute ulnar neuropathy at the elbow. HNPP: hereditary neuropathy with pressure palsy.

The clinical presentation of UNE often results from compression of the ulnar nerve either by direct mechanical compression or by compression of the intrinsic blood supply to the nerve which causes ischemia [1,6,8]. The symptoms of UNE are usually of insidious onset with chronic slow progression [12]. Acute presentation is much less common and often results from focal trauma; it may also result from direct pressure on the nerve at the RTC groove and during prolonged elbow flexion (Figure 1) [13]. There is a paucity of literature on the topic of acute UNE, and it is mainly comprised of case reports [12,14]. In this paper, we describe a large cohort of patients with acute-onset UNE with the primary objectives of determining the underlying etiology and the accompanying EDX and US findings.

## 2. Materials and Methods

Under an Institutional Review Board (IRB)-approved protocol, we performed a 25-year (January 2010–April 2026) retrospective analysis of patients who were referred to our Neurodiagnostic Center for EDX studies in whom the history and clinical examination indicated UNE. Our American Association of Neuromuscular and Electrodiagnostic Medicine (AANEM)-accredited Neurodiagnostic Center evaluates approximately 1500 patients annually referred for EDX studies primarily by hand surgeons, orthopedic surgeons, and neurosurgeons. We included all patients diagnosed with UNE based on clinical evaluation as well as EDX and US studies. The same electromyographer, board-certified in electrodiagnostic medicine and clinical neurophysiology, performed the evaluations of all patients which negated any inter-examiner reliability bias.

### 2.1. Inclusion and Exclusion Criteria

The inclusion criteria were (1) patients whose symptoms of UNE reached a peak intensity within 24 h (waking up with symptoms or noticing the symptoms soon after an injury or after undergoing a medical or surgical procedure) and (2) patients who experienced acute sensory loss and/or muscle weakness in the ulnar nerve distribution without antecedent trauma as well as EDX evidence of localization of ulnar nerve involvement to the elbow. Patients were excluded if they had evidence of a C8 or T1 radiculopathy, brachial plexopathy, polyneuropathy or multi-mononeuropathy, and ulnar neuropathy localized proximal or distal to the elbow.

### 2.2. Metrics Collected

The demographics, clinical findings, EDX findings (focal demyelination, conduction block, loss or decrease in amplitude of sensory nerve action potentials [SNAP], denervation of muscles and severity grade), and US findings were collected. The clinical, EDX, and US data were analyzed to determine the severity of ulnar neuropathy, the underlying pathophysiology (demyelination, conduction block, and axon loss), and the possible etiology.

The clinical metrics collected included (1) clinical signs of ulnar neuropathy (Wartenberg, Pollock, Duchenne, Froment, and Palmaris brevis sign), (2) muscle strength (abductor digiti minimi [ADM], first dorsal interosseous [FDI], other interossei, flexor digitorum profundus—ulnar [FDPu], and FCU), (3) sensory loss, (4) tenderness/positive Tinel sign, and (4) presence or absence of signs of injury or masses along the course of the ulnar nerve.

The EDX studies were performed based on AANEM guidelines using the standard protocol in our lab [15]. A motor nerve conduction study was done by placing recording electrodes over the FDI, ADM, and, if indicated, FCU muscles and stimulating the ulnar nerve at the wrist, distal and proximal to the elbow, as well as the proximal upper arm. Short segment stimulation across the elbow was performed in the presence of significant conduction block for more precise localization. Sensory conduction in both the digital and the dorsal cutaneous branches was studied. Antidromic sensory conduction study was performed by stimulation at the wrist and recording from the ring and little fingers. In most cases, sensory conduction in the dorsal cutaneous branch was also studied by stimulating at the distal forearm and recording from the ulnar side of the dorsum of the hand. Localization to the elbow was based on motor conduction studies. Needle EMG was done using a monopolar needle electrode to document denervation and reinnervation changes.

The US study of the ulnar nerve was conducted with a GE LogiQ machine (GE Healthcare, Boston, MA, USA) using 6–15 or 8–18 MHz probes. The ulnar nerve was studied in its course from the upper arm to Guyon’s canal. The CSA and the diameter of the ulnar nerve were measured at the medial epicondyle as well as 2 cm proximally and 2 cm distally [10]. The upper normal limit of the CSA at the elbow was 10 mm^2^. The long (longitudinal)-axis and short-axis images were obtained. Changes in echogenicity and fascicular morphology were also documented.

Clinical grading was based on the McGowan classification (Table 1) [16,17]. EDX grading was based on the data collected from nerve conduction and EMG studies.

### 2.3. Institutional Review Board Approval

This study was conducted according to the guidelines of the Declaration of Helsinki and was approved by the WCG Institutional Review Board. The IRB determined that our study was exempt under 45 CFR 46.104(d4). The IRB number is 20253566, and the Ethic Approval Code is 09152025. The IRB approval date was 15 September 2025.

## 3. Results

### 3.1. Demographics and Clinical Findings

Among 27,487 patients who underwent EMG studies, 2262 were diagnosed with UNE. Of these, a total of 103 patients were diagnosed with acute UNE at our Neurodiagnostic Center over the duration of this study (Table 2). The mean age was 59.3 years (range: 19–86 years), and the majority of patients were male (74 [71.8%]). The symptomatic side was the right in 53 (51.4%) patients, left in 47 (45.6%), and bilateral in 3 (2.9%). A total of 27 (26.2%) patients complained of elbow pain, and paresthesia was experienced by all patients. Decreased pinprick sensation was noted in 99 (96.1%). Weakness of the following muscles was detected: ADM (94 [91.3%]), FDI (91 [88.3%]), and FDPu (76 [73.8%]). The Wartenberg sign was observed in eight (7.8%) patients, and the Duchenne sign was noted in seven (6.8%). Most patients had a clinical severity grade of 3 (93 [90.3%]).

### 3.2. Etiology

Perioperative injuries were the most common etiology of acute UNE, occurring in 65 (63.1%) patients (Table 3). The most frequent perioperative injury was noted after shoulder surgery in 23 (35.4%) patients, followed by coronary artery bypass (9 [13.8%]) and knee surgery (8 [12.3%]). Eleven (10.7%) patients sustained non-iatrogenic injuries. Based on clinical evaluation, no specific etiology was noted in 27 (26.2%) patients with acute UNE. In one of these patients, surgery showed a Schwannoma with bleeding.

### 3.3. Electrodiagnostic Findings

Focal demyelination was observed by EDX studies in 95 (92.2%) patients, and a conduction block was detected in 24 (23.3%) (Table 4). Motor and sensory axonal involvement was identified in 81 (78.6%) and 86 (83.5%) patients, respectively.

### 3.4. Ultrasound Study Findings

**Figure 2:**
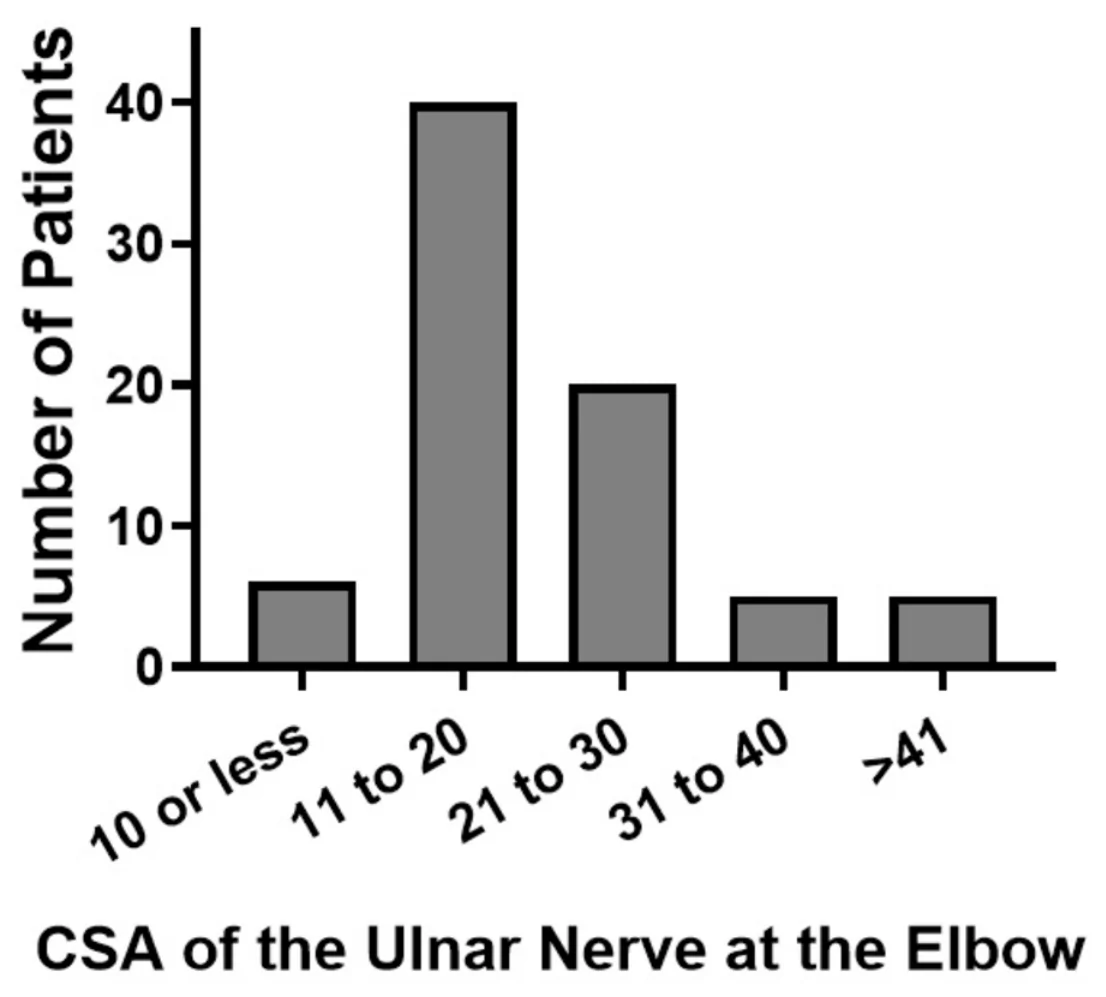
Cross-sectional area of the ulnar nerve at the level of the medial epicondyle in patients presenting with acute ulnar neuropathy at the elbow.

A total of 76 (73.8%) patients underwent a US study (Table 5). Of these 76 patients, 68 (89.5%) had an increase in the CSA of the ulnar nerve (Figure 2) and 51 (67.1%) had a hypoechoic ulnar nerve. The most common site of increased CSA was at the medial epicondyle. Several additional findings were detected by US: neuroma in 3 (3.9%), cysts in 12 (15.8%), and a Schwannoma in 2 (2.6%) patients. The term “cyst” denoted a round or oval anechoic structure often with post-acoustic posterior enhancement. Six (7.9%) patients had either a perineural/epineural hyperechoic appearance or enlarged fascicles of the ulnar nerve. The US study was normal in four (5.3%) patients.

Of the 22 patients who had non-traumatic acute UNE, the US demonstrated an enlarged and hypoechoic ulnar nerve in 14 (63.6%) patients, cysts in 9 (40.9%), and a Schwannoma in 2 (9.1%). The US was normal in four (18.2%) patients (Table 6).

## 4. Illustrative Cases

### 4.1. Case 1

A 65-year-old man presented with numbness of the little and ring fingers and weakness of right hand after undergoing sternal split coronary artery bypass surgery 2 months previously. Loss of pinprick sensation was noted over the little and ring fingers and ulnar side of the palm. There was marked weakness of the FDI, ADM, and FDPu muscles and clawing of the little finger. No SNAPs were elicited over the digital and dorsal cutaneous branches of the ulnar nerve, and there were no CMAPs over the FDI or ADM muscles. Denervation of the FDI, ADM, and FCU muscles was observed with no reinnervation. The US study showed an enlarged ulnar nerve at the medial epicondyle.

### 4.2. Case 2

**Figure 3:**
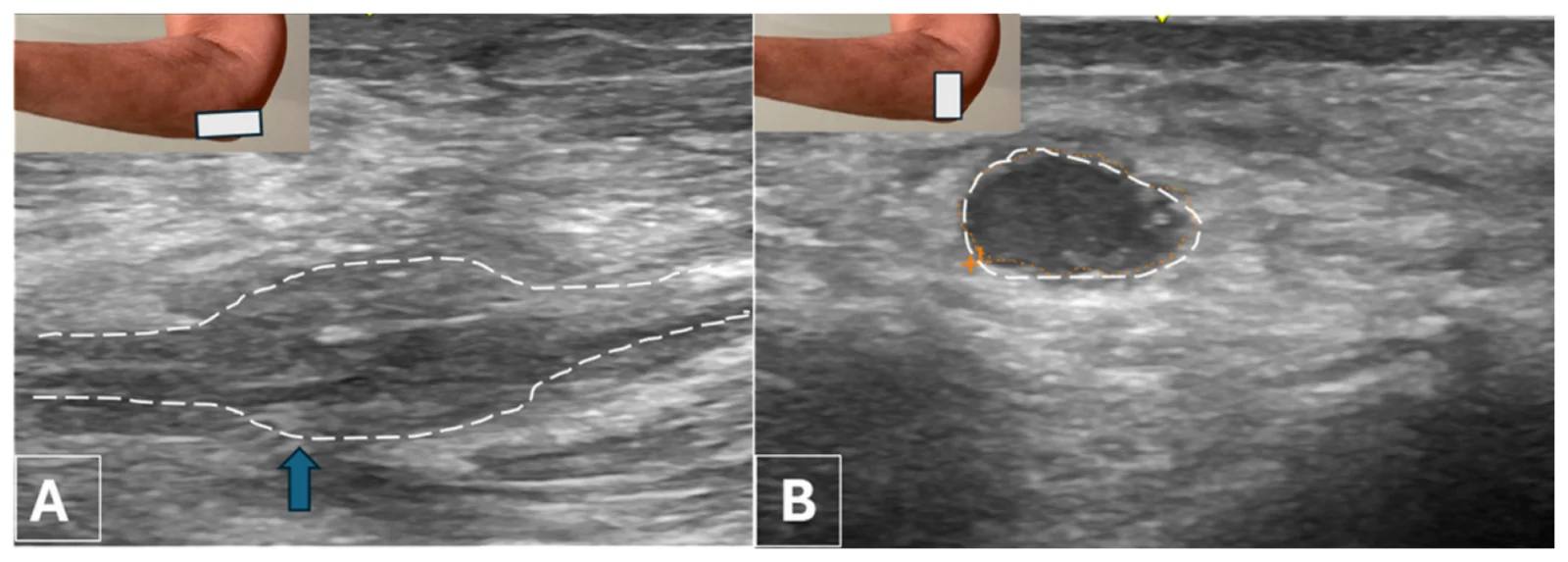
Case #2: Stab injury at the medial side of the elbow resulting in acute ulnar neuropathy. Ultrasound study: (**A**) Long-axis view showing a neuroma in continuity with questionable fascicular continuity (arrow). (**B**) Short-axis view of the ulnar nerve at the medial epicondyle showing an increase in cross-sectional area. The ulnar nerve is hypoechoic.

A 53-year-old man reported persistent numbness and weakness of the right hand beginning immediately after a stab injury to the elbow 3 years earlier. Loss of pinprick sensation over the little finger and medial palm was noted as well as atrophy of the FDI muscle and marked weakness of the FDI, ADM, and FCU muscles. No SNAPs were elicited over the digital and dorsal cutaneous branches, and no CMAPs were detected over the ADM or FDI muscles. Denervation with no reinnervation in the FDI, ADM, or FCU muscles was observed. A neuroma with questionable fascicular continuity was seen in the US study (Figure 3A,B).

### 4.3. Case 3

**Figure 4:**
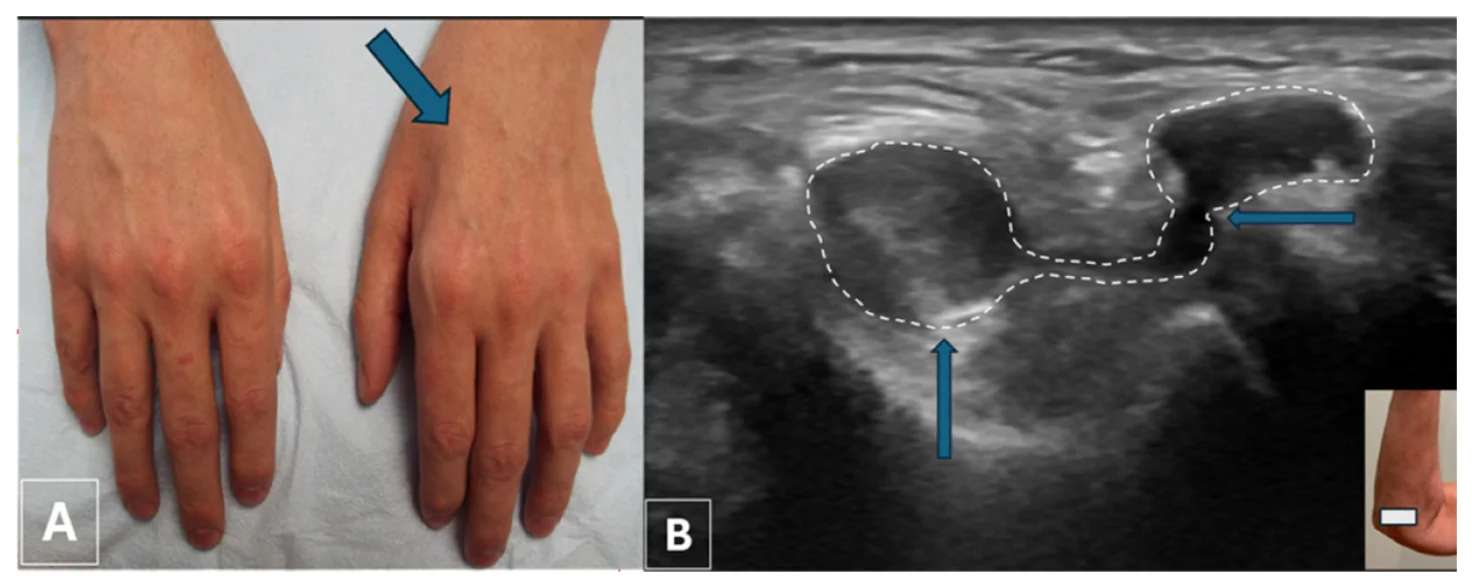
Case #3: (**A**) Arrow points to atrophy of the FDI muscle of the left hand. (**B**) Ultrasound short-axis view at the elbow showing a markedly enlarged ulnar nerve (vertical arrow) from an intraneural cyst, communicating (outlined by a dotted line) with a nearby extraneural cyst (horizontal arrow).

A 45-year-old man described a 4-month history of acute onset of numbness of the left little finger and weakness of muscles without antecedent trauma or unaccustomed activity. Pinprick sensation was lost over the left little finger as well as the palmar and dorsal ulnar side of the hand. Atrophy of the left FDI muscle was seen (Figure 4A). There was also weakness of the left FDI, ADM, FDPu, and FCU muscles. EDX studies demonstrated extreme slowing of motor conduction in the ulnar nerve at the elbow to <10 m/s as compared to the lowest normal of 50 m/s, loss of SNAP in the digital and dorsal cutaneous branches, and denervation of the FDI, ADM, and FCU muscles. The US study showed a cyst compressing the ulnar nerve which had an intraneural component (Figure 4B).

### 4.4. Case 4

**Figure 5:**
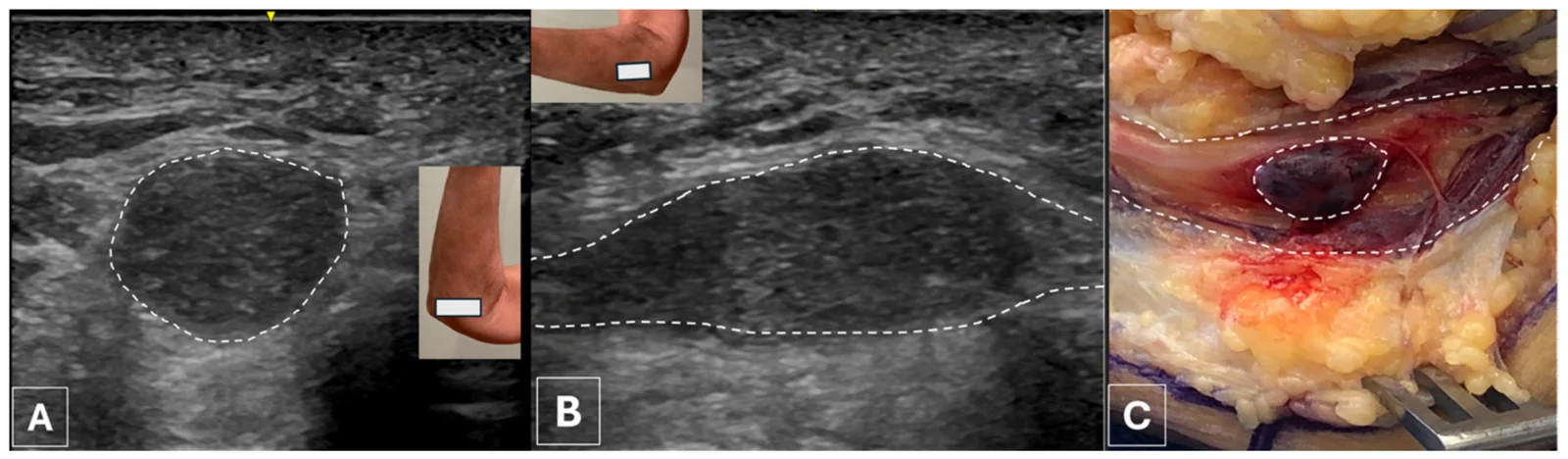
Case #4: (**A**) Ultrasound short-axis view at the elbow showing a markedly enlarged ulnar nerve (dotted line). (**B**) Long-axis view at the elbow showing an oblong mass (dotted line) in the ulnar nerve. (**C**). Intraoperative photo showing a Schwannoma (outer dotted line) with hemorrhage (inner dotted line) inside the mass.

A 69-year-old man reported a 4-week history of sudden onset of pain, numbness, and weakness of the left hand; the numbness involved the little and ring fingers along with pain at the elbow. There was loss of two-point discrimination and light touch over the little and ring fingers with intact pinprick sensation. The FDI, ADM, and FDPu muscles were markedly weak. Focal slowing of motor conduction was observed across the elbow in the left ulnar nerve, and SNAPs were not detected over the digital and dorsal cutaneous branches. There was decreased MUP recruitment and an increase in polyphasic motor units in the ADM and FDI muscles. The US study revealed marked enlargement of the ulnar nerve at the elbow suggestive of a Schwannoma (Figure 5A,B). The postoperative diagnosis was a Schwannoma with hemorrhage (Figure 5C).

## 5. Discussion

Acute UNE may result from blunt trauma, penetrating/lacerating injuries, secondary to supracondylar humeral fractures and elbow joint injuries, and most often from perioperative injuries. In some patients no specific etiology for the acute presentation may be found based on clinical history and EDX findings, but imaging studies (US or MRI) may discover an underlying cause. There are case reports in the literature with a plethora of causes pertaining to spontaneous (non-traumatic/non-iatrogenic) ulnar neuropathy of acute onset, but no large series are available to better understand the incidence and causes.

Perioperative/procedure-related (term preferred over iatrogenic) [18] ulnar neuropathies accounted for the majority of patients presenting with acute onset of symptoms (63%) in this series of 103 cases. Among them, surgical procedures at the shoulder, especially reverse total arthroplasty, were the most common. We have previously reported a series of such cases and speculated on possible mechanisms, including compression of the ulnar nerve at the elbow during surgery or subsequently while immobilized in a sling with the elbow in hyperflexion [19]. Following shoulder surgery, the most common procedures associated with acute ulnar neuropathy at the elbow were coronary artery bypass surgery and knee arthroplasty. In patients undergoing coronary artery bypass surgery, the more common neurologic complication is lower trunk brachial plexus injury from retraction of the split sternum and ulnar nerve compromise at the elbow. There are three theories for the latter, including direct pressure at the retrocondylar location, stretch injury from prolonged flexed position at the elbow, and possible ischemic injury. Wey and Guinn reported that ulnar neuropathy was most common after open-heart surgery and was due to nerve compression at the elbow when the arms were kept at the side [20]. They noted “a substantial reduction in nerve injury was effected by placing the forearm behind the head in the OR”. However, considering the occurrence of UNE accompanying surgical procedures in many different anatomical locations, the common underlying factor is likely to be compression or stretch of the nerve at the retrocondylar area at the elbow. Swenson et al. describe ulnar neuropathy at the elbow even after appropriate padding and positioning and draw attention to ischemic injury as an additional factor [21].

There is a preponderance of male patients in this series who experienced perioperative ulnar neuropathy at the elbow. This pattern has been recognized by others, and multiple explanations have been given. In their study of unembalmed cadavers using US, Contreras et al. noted that the tubercle of the coronoid process of the ulna was significantly larger in the male and predisposed to external compression-induced ischemia “because the nerve and its arterial supply (posterior ulnar recurrent artery) are covered by skin, subcutaneous fat and a very thin aponeurosis of flexor carpi ulnaris” [22]. They also mentioned that a larger fat pad seen in women protected the nerve from external compression [22]. Another factor is the larger muscle tissue in males that predisposes to more compression under FCU aponeurosis [23].

Among the spontaneous (non-trauma) group, it was noted that two patients drove long distances with the elbow resting on the windowsill and another one had chopped firewood for several hours prior to symptom onset. It is possible that the ulnar nerve was compressed or stretched at the retrocondylar groove and/or the cubital tunnel sufficiently to cause the acute onset of symptoms in those patients.

Of 12 patients in this study who had cysts in the vicinity of the ulnar nerve at the elbow, 9 were in the non-trauma/perioperative group. There are a number of reports of cysts causing symptoms of CBTS [24,25,26,27,28]. Acute onset of symptoms in the presence of a cyst has been only rarely reported, mostly with the occurrence of acute foot drop from a ganglion cyst in relation to the common fibular nerve [29,30]. There is one case report of acute ulnar nerve palsy from a ganglion cyst at Guyon’s canal [31]. These authors postulate that “acute protrusion or growth of the ganglion cyst, which wrist movements might have provoked, accounted for the compression of [the patient’s] ulnar nerve’s branch.” In our cases, the precise mechanism of the acute onset of symptoms of UNE in the presence of cystic lesions is unclear. A rapid increase in the size of the cyst could cause direct pressure or ischemia facilitating acute compromise of nerve function [32]. Another possibility is sudden intraneural extension of a pre-existing cyst through one of the articular branches of the ulnar nerve.

This study underscores the importance of US as an essential step in evaluating patients presenting with UNE with no history of trauma, preceding surgical procedures under general anesthesia, or other causes of extraneous compression of the nerve at the elbow. In cases of injury, US may be useful in distinguishing axonotmesis from neurotmesis and detecting neuromas with and without fascicular continuity.

### Strengths and Limitations

The strength of the present study is that it features a large number of patients with acute onset UNE and highlights the major causes, of which perioperative injury is the single most common cause. Most of the periprocedural ulnar neuropathies resulted from not protecting the nerve with adequate padding or a stretch injury at the elbow during prolonged procedures anywhere in the body. We also describe the important role of both EDX and US studies in localizing ulnar neuropathies to the elbow and grading of the severity and underlying pathophysiology of neuropathy. Furthermore, we stress the important role of US studies, available at the point of care, in determining the underlying etiology. Limitations of this study include its retrospective nature and lack of follow-up as most patients were only evaluated on one occasion at our Neurodiagnostic Center.

## 6. Conclusions

In this study, we sought to determine the etiology of acute UNE using clinical evaluation as well as EDX and US studies. The most common cause of acute UNE was periprocedural injury during several surgical procedures at many different anatomical locations, most frequently following shoulder surgery. Occasionally, patients may develop acute UNE without a history of antecedent trauma or surgical procedure. EDX and US studies may provide complementary data to localize, grade, and unravel the cause of acute UNE and guide management.

## Figures and Tables

**Table 1:** Clinical and electrodiagnostic grading of acute ulnar neuropathy at the elbow (clinical grading based on the McGowan classification [16]).

Grading	Mild	Moderate	Severe
Clinical	Sensory symptoms only, decreased pinprick sensation over the little and ring fingers	Sensory abnormalities, weakness of the ADM, FDI, and FDPu muscles with no atrophy	Sensory abnormalities and weakness and atrophy of the FDI and ADM muscles
Electrodiagnostic	Focal slowing [MNCV of <49 m/s] or a drop in MNCV > 10 m/s across the elbow, normal SNAP over the little finger on stimulation at the wrist, and normal EMG of the FDI and ADM muscles	Focal slowing with or without a motor conduction block (50% or more drop in amplitude of CMAP between stimulation below the elbow vs. above the elbow), absent or low amplitude SNAP, and needle EMG demonstrating a decrease in motor unit recruitment and an increase in polyphasic MUPs	Low amplitude or absent CAMP, absent SNAP, and denervation of the FDI and ADM muscles

ADM: abductor digiti minimi. FDI: first dorsal interosseous. FDPu: flexor digitorum profundus—ulnar. MNCV: motor nerve conduction velocity. SNAP: sensory nerve action potentials. EMG: electromyography. MUP: motor unit potential. CAMP: compound muscle action potential.

**Table 2:** Demographics and clinical findings of acute ulnar neuropathy at the elbow.

Metrics	Number of Cases (*n* = 103)
Age	Mean: 59.3 years (Range: 19–86 years)
Gender	
Male	74 (71.8%)
Female	29 (28.2%)
Side of symptoms	
Right	53 (51.4%)
Left	47 (45.6%)
Bilateral	3 (2.9%)
Elbow pain	27 (26.2%)
Paresthesia	103 (100.0%)
Decreased pinprick sensation	99 (96.1%)
Muscle weakness	
ADM	94 (91.3%)
FDI	91 (88.3%)
FDPu	76 (73.8%)
Clinical grade	
3 (severe)	93 (90.3%)
2 (moderate)	9 (8.7%)
1 (mild)	1 (0.97%)

ADM: abductor digiti minimi. FDI: first dorsal interosseous. FDPu: flexor digitorum profundus—ulnar.

**Table 3:** Acute ulnar neuropathy at the elbow from trauma.

Etiology	Number of Cases (*n* = 103)
Perioperative/procedural injuries	65 (63.1%)
Shoulder	23 (35.4%)
Coronary artery bypass	9 (13.8%)
Knee	8 (12.3%)
Elbow	3 (4.6%)
Others *	22 (33.8%)
Other injuries	11 (10.7%)

* Lumbar spine: 3; cervical spine: 2; prostate/bladder: 2; colon: 1; aortic valve: 1; contraceptive implant: 1; CMC joint repair with sling: 1; hip: 1; kidney: 1; liver transplant: 1; thenar cyst removal: 1; mammogram: 1; intravenous infusion at antecubital fossa: 1; laparotomy: 1; coma: 1; prolonged chronic congestive heart failure/hospital stay/restraints: 3.

**Table 4:** Electrodiagnostic findings of acute ulnar neuropathy at the elbow.

Metrics	Number of Cases (*n* = 103)
Focal demyelination	95 (92.2%)
Conduction block	24 (23.3%)
Motor axonal involvement	81 (78.6%)
Sensory axonal involvement	86 (83.5%)

**Table 5:** Ultrasound findings of acute ulnar neuropathy at the elbow.

Ultrasound Findings	Number of Cases (*n* = 76)
Increase in cross-sectional area	68 (90.6%)
Hypoechoic ulnar nerve	51 (68.0%)
Neuromas	3 (5.3%)
Cysts	12 (15.8%)
Tumors	2 (2.6%)
Others *	6 (8.0%)

* Others: either a perineural/epineural hyperechoic appearance or large fascicles of the ulnar nerve.

**Table 6:** Ultrasound findings of non-traumatic acute ulnar neuropathy at the elbow.

Findings	Number of Cases (*n* = 22)
Enlarged and hypoechoic ulnar nerve	14 (63.6%)
Cysts	9 (40.9%)
Schwannoma	2 (9.1%)
Normal	4 (18.2%)

## Data Availability

All of the data for this study is included in the current article.
